# Acid Tests and the Hope for Adequate Oxygen Intake in 2021

**DOI:** 10.1093/function/zqaa035

**Published:** 2020-12-02

**Authors:** Ole H Petersen

**Affiliations:** School of Biosciences, Cardiff University, Wales, UK

The year 2020 will undoubtedly, and for an obvious reason, always retain a special place in the history books. It is now clear that it was not the easiest year to start *FUNCTION*, but the decision to do so was made in 2019, long before anyone knew about COVID-19. The pandemic caused significant problems for *FUNCTION*’s launch plans, which had been closely linked to physical meetings, including EB2020. These meetings were of course all eventually canceled. Nevertheless, as described in some detail by the APS President, Linda Samuelson,^1^ *FUNCTION* has moved from concept to reality with a launch in 2020 that has been remarkably successful. I am profoundly grateful to the executive editors and editorial board members who have shown real confidence in the journal by submitting high-quality articles and also to the growing number of authors outside the editorial board who have sent excellent papers to us. I want to acknowledge here the enormous amount of hard and effective work carried out by our Managing Editor, Dr. Chris England. Without his enthusiasm and drive, we would not have done nearly as well as we have. The weekly zoom meetings with Chris, Christina Bennett, and Nina Burdakova have been focal points for me this year and been the principal vehicle by which we have made key decisions about the journal.

At the end of a year, it is natural to look back not only on what was achieved but also on what we have lost. Sadly, several giants in the physiological sciences passed away. Here I shall mention a few I had the privilege to know personally, who worked in the field of ion transport mechanisms. Sir Michael Berridge FRS died on February 13, 2020. I met him for the first time 50 years ago at a Royal Society meeting in London, at which we both gave our first talks at this august institution. His most important discovery was the identification of inositol 1,4,5-trisphosphate (IP_3_) as an important intracellular messenger molecule.^2^ Together with Irene Schulz (see below), he showed that this molecule was the long sought after agent responsible for mediating the release of Ca^2+^ from the endoplasmic reticulum (ER) elicited by neurotransmitters or hormones.^3^ For a more detailed evaluation of Mike’s work and influence, I refer to the obituary published earlier this year in *Cell Calcium*.^4^ The funeral service, held at Trinity College Chapel in Cambridge, England on March 2—just before COVID-19 would make such occasions impossible—was a dignified and ultimately uplifting celebration of Mike’s immense influence on a key area of cellular physiology.

Gerhard Giebisch, who passed away on April 6, was another scientific giant I met in 1970. Gerhard chaired what was for me a very important meeting on “Electrophysiology of Epithelial Cells” at Schloss Rheinhartshausen, at the Rhine river in Germany. It was a closed meeting with a small number of invited speakers, including Hans Ussing, Jared Diamond, Karl Ullrich, and Eberhard Frömter, providing unusual opportunities for real in-depth discussions. Gerhard was a wonderful host, leading the discussions in his characteristically elegant, but also incisive manner. Over many years I had valuable interactions with Gerhard at many meetings in Europe, the United States, and Japan. He dominated the field of kidney physiology and most of our knowledge about the handling of K^+^ in the kidney is derived from his work. He always emphasized the importance of understanding the function of the organ in the context of the homeostasis of the whole body and would therefore undoubtedly have been a strong supporter of our journal. For more details about Gerhard’s scientific work, I recommend Peter Aronson’s excellent obituary.^5^

Writing this editorial in November 2020, I take the liberty of incorporating the last 2 months of 2019 into these end-of-year thoughts, as this allows me to pay tribute to yet another giant of physiology. George Sachs passed away on November 12, 2019. He received the Canada Gairdner International Award in 2004 “for his elucidation of mechanisms of gastric acid secretion and the development of novel therapies for gastric disease.” He was instrumental in the development of omeprazole, one of the most widely used and safest drugs ever produced, which is daily helping millions of people across the globe, suffering from peptic ulcer disease and Gastro-Esophageal Reflux Disease (GERD), to live a normal painless life. When I was a medical student in Copenhagen in the 1960s, the most frequent gastrointestinal disease requiring hospitalization was peptic ulcer disease. The treatment, partial gastrectomy and/or cutting the vagal nerve supply to the stomach, was only partially successful and often led to major complications and real misery for the patients. James Black’s development of cimetidine, which is an effective histamine (H2) antagonist, certainly improved the treatment of gastric acid-related disorders, but was not fully effective. One reason is that there are three different agents eliciting acid secretion from the oxyntic cells in the stomach, namely acetylcholine, gastrin, and histamine. Blocking the histamine H2 receptors therefore only deals with one of the pathways and compensatory increases in the stimulation by the other two agents can occur. George Sachs discovered that the crucial active step in acid secretion is the operation of the H^+^/K^+^-ATPase, which he and his collaborators characterized in great detail. As one of the invited speakers at the symposium on “Hydrogen Ion Transport in Epithelia,” hosted by Irene Schulz in Frankfurt, Germany, in the summer of 1980, I witnessed no less than five presentations by George and his collaborators on the gastric H^+^/K^+^-ATPase. In one of these talks, the first results demonstrating the specific inhibition of this enzyme by substituted benzimidazoles were shown. The data on the inhibitory action of agent H149/94, later named picoprazole (developed by AB Hässle in Sweden, the research arm of what was then known as Astra—later AstraZeneca), were published the following year.^6^ A further refinement led to H168/68, known as omeprazole, which was then launched in Europe in 1988 (Losec) and in the United States in 1990 (Prisolec). This was the real revolution in the treatment of peptic ulcer disease and in the late 1990s omeprazole became the world’s best-selling drug. A further refinement resulted in the production of esomeprazole (Nexium), which was launched in 2000. This drug can now be found on the shelves of supermarkets around the world and can be bought without even talking to a pharmacist. It is extremely effective and safe and represents the ultimate triumph of treating a disease that used to cause debilitating pain, preventing a normal life.

Everyone who knew George would say that he was the most intelligent man they had ever met. I remember vividly a meeting at King’s College, London, at a time when I was wrestling with a difficult problem of data interpretation, where I had the opportunity on the very same day to ask three outstanding world-leading investigators exactly the same question. The only one who instantly understood the problem and was able to provide effective advice was George. He was a formidable opponent in discussions and it was therefore with considerable trepidation that I accepted an invitation to engage in a “scientific duel” with him at a large gastroenterology meeting in Hamburg. I was to “defend” the electrophysiological approach to cellular ion transport studies, whereas George was supposed to advance the case for the use of chemical indicators. Both approaches are obviously valid and useful, but many attendees were clearly hoping for a “knock-out match.” In the event, it was a friendly and scientifically based exchange. Later, after George in 1982 had become Director of the Center for Ulcer Research and Education at UCLA, he generously invited me to give the 1985 Morton I. Grossman Memorial Lecture. By solving the problem of treating a very severe and common disease effectively, George did more for the welfare of mankind than any other physiologist working in the 20th century. His work certainly passed the acid test.

Having mourned the loss of these three giants of physiology, it is a pleasure to look forward to the 80th birthday of Irene Schulz in January 2021. While still a medical student at the Free University in Berlin, Irene pioneered—together with her mentor Karl Ullrich—micropuncture studies of exocrine glands and published the very first data on the composition of the primary secretion in the secretory coil of sweat glands.^7^ These studies provided the first direct evidence for the two-step hypothesis of sweat formation, in which the coil produces a solution with plasma-like Na^+^ and Cl^−^ concentrations and the duct reabsorbs NaCl without water, explaining the relatively low Na^+^ and Cl^−^ concentrations in the final sweat. Because the electrical potential in the duct lumen was negative with respect to the interstitial fluid, it was concluded that the duct actively reabsorbed Na^+^ with Cl^−^ following passively.^7^ After graduation, and during a stay at the NIH, Irene extended these studies to sweat glands in cystic fibrosis (CF) patients and showed that the primary secretion was unaffected by CF.^8^ As the NaCl concentration in the final sweat of these patients is much higher than normal, she concluded that there was a defect of salt absorption in the duct. Because the negative potential in the duct lumen was undiminished in CF, Irene proposed that the defective salt reabsorption was due to a reduced Cl^−^ permeability in the duct epithelium.^8^ This was in fact the discovery of the fundamental problem in CF, namely the abnormal and defective Cl^−^ channel function. When Karl Ullrich was appointed Director of the Max Planck Institute for Biophysics in Frankfurt, Irene became one of the Group Leaders in the Institute. As a trustee and member of the Institute’s Advisory Board for 15 years, I was able to follow Irene’s research work which, like mine, became focused on the role of Ca^2+^ in the control of exocrine pancreatic secretion. Happily, our scientific interactions continued after Irene’s appointment to the Chair of Physiology at the University of the Saarland in 1991. Irene’s greatest success, and the work she is best known for, came about as a result of the collaboration with Mike Berridge in Cambridge that, as mentioned above, proved that IP_3_ is the intracellular messenger that opens Ca^2+^ channels (IP_3_ receptors) in the ER membrane and thus initiates the Ca^2+^ signal that stimulates the secretory process. The paper reporting these data remains a landmark paper in the field of Ca^2+^ signaling.^3^^,^^4^

I shall be chairing an Academia Europaea (AE) webinar on “New aspects on COVID-19 on January 26, 2021, which is the day after Irene’s 80th birthday and shall use this occasion for a warm congratulation message to a friend and colleague I have known since we first met at a conference in Philadelphia in 1968. The AE webinar features talks by several *FUNCTION* editors,^9^ who have published on the pathophysiology of COVID-19 in our journal, including a presentation by José López-Barneo that may explain the pathophysiological mechanism underlying the silent hypoxemia in severe cases of this disease.^10^ The acid tests for the enormous amounts of work on COVID-19 that have already been carried out by the biomedical science community in 2020 will of course come in 2021. My hope for the New Year is that we shall all be able to take in sufficient amounts of oxygen.

## Funding

The author acknowledges support from the European Commission’s Horizon 2020, grant agreement 737432.

## Conflict of Interest Statement

None declared.
